# Blocking a vicious cycle nNOS/peroxynitrite/AMPK by S-nitrosoglutathione: implication for stroke therapy

**DOI:** 10.1186/s12868-015-0179-x

**Published:** 2015-07-15

**Authors:** Mushfiquddin Khan, Tajinder S Dhammu, Fumiyo Matsuda, Avtar K Singh, Inderjit Singh

**Affiliations:** Department of Pediatrics, Medical University of South Carolina, Charleston, SC 29425 USA; School of Health Science, Kagoshima University, Kagoshima, Japan; Department of Pathology and Laboratory Medicine, Medical University of South Carolina, Charleston, SC USA; Ralph H. Johnson VA Medical Center, Charleston, SC USA

**Keywords:** AMP-activated protein kinase, Neuronal nitric oxide synthase, Neuroprotection, Peroxynitrite, S-nitrosoglutathione, Stroke, Cerebral ischemia reperfusion

## Abstract

**Background:**

Stroke immediately sets into motion sustained excitotoxicity and calcium dysregulation, causing aberrant activity in neuronal nitric oxide synthase (nNOS) and an imbalance in the levels of nitric oxide (NO). Drugs targeting nNOS-originated toxicity may therefore reduce stroke-induced damage. Recently, we observed that a redox-modulating agent of the NO metabolome, S-nitrosoglutathione (GSNO), confers neurovascular protection by reducing the levels of peroxynitrite, a product of aberrant NOS activity. We therefore investigated whether GSNO-mediated neuroprotection and improved neurological functions depend on blocking nNOS/peroxynitrite-associated injurious mechanisms using a rat model of cerebral ischemia reperfusion (IR).

**Results:**

IR increased the activity of nNOS, the levels of neuronal peroxynitrite and phosphorylation at Ser^1412^ of nNOS. GSNO treatment of IR animals decreased IR-activated nNOS activity and neuronal peroxynitrite levels by reducing nNOS phosphorylation at Ser^1412^. The Ser^1412^ phosphorylation is associated with increased nNOS activity. Supporting the notion that nNOS activity and peroxynitrite are deleterious following IR, inhibition of nNOS by its inhibitor 7-nitroindazole or reducing peroxynitrite by its scavenger FeTPPS decreased IR injury. GSNO also decreased the activation of AMP Kinase (AMPK) and its upstream kinase LKB1, both of which were activated in IR brain. AMPK has been implicated in nNOS activation via Ser^1412^ phosphorylation. To determine whether AMPK activation is deleterious in the acute phase of IR, we treated animals after IR with AICAR (an AMPK activator) and compound c (an AMPK inhibitor). While AICAR potentiated, compound c reduced the IR injury.

**Conclusions:**

Taken together, these results indicate an injurious nNOS/peroxynitrite/AMPK cycle following stroke, and GSNO treatment of IR inhibits this vicious cycle, resulting in neuroprotection and improved neurological function. GSNO is a natural component of the human body, and its exogenous administration to humans is not associated with any known side effects. Currently, the FDA-approved thrombolytic therapy suffers from a lack of neuronal protective activity. Because GSNO provides neuroprotection by ameliorating stroke’s initial and causative injuries, it is a candidate of translational value for stroke therapy.

## Background

Ischemic stroke (almost 87% of all strokes) and transient ischemic attack (TIA) obstruct oxygen and nutrient supply to the brain. Instantaneously, the obstruction of blood supply causes necrotic neuronal death in the injury core; however, even greater apoptotic neuronal damage is caused by nitroxidative stress and neuroinflammation in the cortical penumbra and hippocampal areas [1]. Neuronal nitric oxide synthase (nNOS)-derived nitroxidative stress occurs due to calcium homeostasis derailment, excitotoxicity, and an imbalance in the nitric oxide (NO) metabolome. These redox-based injuries later hamper functional recovery [2, 3]. Other than thrombolysis by tissue plasminogen activator, which offers only a short window of treatment (~3–4 h) beyond which severe damage results, an effective neuroprotective stroke therapy is not available mainly due to limited understanding of the initial nitroxidative signaling mechanisms of the disease [1].

nNOS contributes approximately 90% to NOS activity in normal rodents [4]. Of the three known nitric oxide synthases, nNOS activity plays a critical role in neuronal cell death during the acute ischemia reperfusion (IR) phase [5]. Inhibition of nNOS activity following IR [5, 6] has been shown to be neuroprotective and nNOS KO mice show reduced infarct volume [4, 7–10], indicating that nNOS plays a significant role in IR injury. Like nNOS, endothelial nitric oxide synthase is also aberrantly activated early after stroke. However, endothelial nitric oxide synthase is localized primarily in endothelium and endothelial nitric oxide synthase -derived NO, via peroxynitrite, has been associated with blood–brain barrier leakage, cerebral hemorrhage and edema [11–13]. In rodent, inducible nitric oxide synthase is expressed from ~12 h to several days after IR and its immunoreactivity is present mainly in inflammatory cells and endothelium [14–16]. Therefore, the focus of this acute IR study is to investigate the mechanisms of nNOS regulation for developing stroke therapy.

Recent reports document that S-nitrosylation of nNOS regulates its activity [11, 17], in addition to phosphorylation/dephosphorylation [18]. In resting neurons, nNOS is inhibited mainly by S-nitrosylation of Cys^331^; however, immediately following stroke injury, nNOS is activated by NMDA receptor-mediated excitotoxicity and a sustained calcium influx through site-specific phosphorylation (Ser^1412^) and denitrosylation (Cys^331^). In such an environment, nNOS-derived NO is converted to peroxynitrite by an instantaneous diffusion-limited reaction with superoxide [19]. Peroxynitrite can activate AMPK via the activation of upstream AMPK kinase LKB1, thus maintaining a vicious cycle of its own production [20].

The interplay between nNOS and neuronal AMPK during the acute phase of stroke is now recognized to contribute to neuronal loss [21]. AMPK is a cellular energy sensor and an important potential target for stroke treatment. However, the timing, duration and degree of its activation are critical for the outcome of stroke injury [21, 22]. AMPK is activated during decreased cellular energy supply (AMP vs. ATP ratio). It is highly expressed in neurons (AMPKα2) and is rapidly activated in an energy-deprived status such as that which follows stroke [23]. Its activation during the acute phase of IR is deleterious; indeed, both pharmacological inhibition and gene deletion of AMPK were found to be neuroprotective [23]. AMPK has been reported to phosphorylate nNOS [24, 25]. During acute IR disease with dysregulated calcium flux, AMPK activation possibly keeps nNOS hyperactivated via sustained phosphorylation of Ser^1412^ of nNOS. This phenomenon results in Cys^331^ denitrosylation, leading to sustained peroxynitrite formation and thus peroxynitrite-mediated neuronal loss. In contrast to peroxynitrite, an endogenous signaling molecule of the NO metabolome, S-nitrosoglutathione (GSNO), has been documented to reduce the levels of peroxynitrite as well as neuronal cell death in a number of neurodegenerative diseases, including stroke [11, 26, 27], traumatic brain injury [28, 29] and vascular dementia [30]. It can inhibit the activity of nNOS under excitotoxic conditions via the S-nitrosylation of Cys^331^ of nNOS [17, 18], leading to reduced formation of peroxynitrite.

GSNO executes its action mainly via S-nitrosylation of target proteins [31]. In stroke pathology, the levels of GSNO and the consequent S-nitrosylated proteins are believed to decrease due to four major reasons: (a) decreased oxygen supply under ischemic/hypoxic condition reduces GSNO biosynthesis; (b) excessive superoxide formed during reperfusion instantaneously reacts with nitric oxide synthase (NOS)-derived NO, forming peroxynitrite and thus reducing NO bioavailability for GSNO biosynthesis; (c) biosynthesis of GSNO is decreased as a result of reduced levels of glutathione (redox imbalance) and NO (due to its reaction with superoxide) under IR conditions. Furthermore, NO reacts slowly with glutathione as compared with superoxide [32]; and (d) in the inflammatory environment, the expression of the GSNO-degrading enzyme GSNO reductase is increased [33], leading to reduced levels of GSNO. Therefore, the subject of investigation for this study was the exogenous supplementation of GSNO to down regulate the aberrant activity of nNOS and AMPK, leading to neuroprotection and functional recovery.

The overall goal of the present study is to investigate whether GSNO provides neuroprotection by reducing the levels of peroxynitrite via the inhibition of nNOS activity. Our initial studies show that treatment of IR animals with GSNO reduced phosphorylation at Ser^1412^, possibly via the inhibition of LKB1 and AMPK activities, and thus attenuated nNOS activity and peroxynitrite-mediated brain injury. The GSNO treatment also improved neurological functions.

## Methods

### Reagents

GSNO was purchased from World Precision Instruments (Sarasota, FL) and dorsomorphin dihydrochloride (Compound c) from Tocris Bioscience (Bristol, UK). 5-Amino-1-[(2*R*,3*S*,4*R*,5*R*)-tetrahydro-3,4-dihydroxy-5-(hydroxymethyl)furan-2-yl]-1*H*-imidazole-4-carboxamide (AICAR) and 5,10,15,20-tetrakis(4-sulfonatophenyl) porphyrinato iron (III) (FeTPPS) were obtained from Abcam (Cambridge, UK) and Calbiochem, (La Jolla, CA, USA), respectively. All other chemicals and reagents used were purchased from Sigma-Aldrich (St. Louis, MO, USA), unless stated otherwise.

### Animals

Animals were male Sprague–Dawley rats weighing between 250 and 290 g at the time of surgery. Number of animals used in this study is described in figure legends. All animals received humane care in compliance with the Medical University of South Carolina’s (MUSC) guidance and the National Research Council’s criteria for humane care. Animal procedures were approved by the institutional animal care and use committee of MUSC.

### Experimental groups, drugs and dose

The animals were randomly divided into eight groups: (1) ischemia reperfusion treated with vehicle (IR), (2) IR + GSNO treatment (GSNO), (3) IR + compound c treatment (Comp c), (4) IR + AICAR treatment (AICAR), (5) IR + FeTPPS treatment (FeTPPS), (6) IR + 7-nitroindazole (7-NI) treatment, (7) IR + AICAR + GSNO, and (8) sham-operated treated with vehicle (Sham). The number of animals used in each experiment is indicated in figure legends. In the GSNO treatment group, the rats were administered freshly prepared GSNO (0.25 mg/kg body weight), which was dissolved in sterile saline (~250 μl) and administered intravenously slowly at reperfusion or as stated. While 7-NI (50 mg/kg ip) was administered at the reperfusion, AICAR (500 mg/kg, ip), Comp c (20 mg/kg, ip) and FeTPPS (3 mg/kg, iv) were administered 1 h after reperfusion. The dosage of treatment was based on our previously reported dose response curve study in a rat model of IR or effective dose reported in stroke IR models [11, 23, 34]. Physiological parameters did not alter after the treatments. Details of the study on physiologic parameters in IR rats have been reported earlier [26].

### Middle cerebral artery occlusion rat model

Rats were anesthetized by ketamine hydrochloride (90 mg/kg body weight) and xylazine (10 mg/kg body weight) administered ip. Stroke was induced for 60 min by left middle cerebral artery occlusion using an intraluminal filament as previously described [26, 35]. To ensure the obstruction, regional cerebral blood flow was monitored during the occlusion and early reperfusion [26]. Reperfusion was established by withdrawal of the filament. After specified time points, animals were sacrificed with an overdose of ketamine hydrochloride.

### Evaluation of ischemic infarct

Coronal sections (2 mm) were immersed in 1% solution of TTC in phosphate-buffered saline (PBS, pH 7.4) at 37°C for 15 min, as described previously [36]. After staining, infarctions were measured using Scion Image software (Scion Corp., Frederick, MD, USA). Total infarct area was multiplied by the thickness of the brain sections to obtain infarct volume, as described previously [27]. In order to minimize the error introduced by edema and liquefaction after infarction, an indirect method for calculating infarct volume was used [35, 37]. The non-infarcted area in the ipsilateral hemisphere was subtracted from that in the contralateral hemisphere, and infarct volume was calculated using the following formula: corrected percentage of infarct volume = (contralateral hemispheric volume − ipsilateral non-infarcted volume)/contralateral hemispheric volume.

### Evaluation of neurological score

Neurological deficits were evaluated by an observer blinded to the identity of the groups. A neurological grading system with a 4-point scale (0–3), as described previously [27], was used: 0, no observable neurological deficit (normal); 1, failure to extend right forepaw on lifting the whole body by tail (mild); 2, circling to the contralateral side (moderate); 3, leaning to the contralateral side at rest or no spontaneous motor activity (severe). The animals not showing paralysis at 1 h after middle cerebral artery occlusion were excluded from the study because the reduction of blood flow may not have produced an infarction of adequate size to cause quantifiable neurological deficits in those animals.

### Histological evaluation

At hour 4 post-IR, the animals were sacrificed and perfused before the brain was fixed in 10% formalin. The tissue was processed for histology and immunohistochemistry (IHC) as previously described [36]. The brain sections (8 µM thick) were stained as described [36] with Nissl, Bielschowsky’s silver, H&E. The histological analysis was performed in the penumbra area by an investigator who was blinded to the experimental group. While Nissl staining determines neuronal cell damage, H&E indicates overall tissue damage. Bielschowsky’s silver staining provides indication of the loss of axonal integrity.

### Immunohistochemistry (IHC) analysis

Paraffin-embedded sections from the formalin-fixed brain tissues were stained for 3-NT (Abcam Cat# ab7048, RRID:AB_305725) and NeuN (Merck Cat# MAB377, RRID:AB_11210778), as described previously [29]. In brief, the brain tissue sections were deparaffinized, sequentially rehydrated in graded alcohol, and then immersed in PBS (pH 7.4). Slides were then microwaved in antigen unmasking solution (Vector Labs), cooled and washed 3 times for 2 min in PBS. Slides were then blocked by 5% non-fat dry milk in PBS for 20 min at room temperature. Sections were incubated overnight with antibodies of 3-NT and NSE. They were then rinsed 3 times for 5 min in PBS containing 0.1% Tween-20. Secondary anti-mouse IgG, conjugated with Texas red (Vector Laboratories Cat# TI-2000, RRID:AB_2336178) for 3-NT and secondary anti-rabbit IgG, conjugated with FITC (Vector Laboratories Cat# FI-1000, RRID:AB_2336197) for NSE were incubated on slides for 60 min. All sections were examined for immunoreactivity in the penumbra area using an Olympus microscope equipped for epifluorescence and DP Controller software. Figures were compiled in Adobe Photoshop software.

For double labeling, sections were incubated first with an antibody of 3-NT followed by NSE. For immunofluorescent double-labeling, immune complexes were visualized with anti-mouse IgG (Vector Laboratories Cat# TI-2000, RRID:AB_2336178) and anti-rabbit IgG (Vector Laboratories Cat# FI-1000, RRID:AB_2336197). In another set of experiments, sections were also incubated with FITC or Texas Red conjugated IgG without the primary antibody as a negative control to confirm the specificity of the primary antibody. Slides were examined for immunofluorescence using an Olympus microscope equipped with an epifluorescence filter and Adobe Photoshop software [29].

### Western blot analysis

Western blot was performed using fresh or frozen tissue, as previously described [11]. The following antibodies were used; phospho-Thr^172^-AMPKα (Cell Signaling Technology Cat# 2535S RRID:AB_331250), AMPKα (Cell Signaling Technology Cat# 2532S RRID:AB_10694064), phospho-Ser^79^-ACC Cell Signaling Technology Cat# 3661S RRID:AB_330337) and phospho-Ser^428^-LKB1 (Cell Signaling Technology Cat# 3482S RRID:AB_2198321), nNOS (Abcam Cat# ab1376 RRID:AB_300614), phospho nNOS Ser^1417^, equivalent to human Ser^1412^ (Abcam Cat# ab90443 RRID:AB_2049208) and β-actin (Sigma-Aldrich Cat# A3853 RRID:AB_262137), followed by horseradish peroxidase-conjugated, goat anti-rabbit secondary antibody (Jackson ImmunoResearch Cat# 111-035-045 RRID:AB_2337938). Protein concentrations were determined using protein assay dye from Bio-Rad Laboratories (Hercules, CA). Densitometry of protein expression was performed using a GS800 calibrated densitometer from Bio-Rad laboratories (Hercules, CA).

### NOS activity assay

NOS activity in the penumbra brain area was determined by the conversion of L-[4,5-^3^H] arginine (American Radiolabeled, Inc., St. Louis, MO, USA) to L-[4,5-^3^H] citrulline in the presence or absence of the competitive NOS inhibitor L-NAME using an NOS assay kit from Cayman Chemicals (Ann Arbor, MI) and following the instructions described therein. Total cNOS activity was determined by subtracting calcium independent NOS activity. The nNOS inhibitor, 7-NI (50 mg/kg), and the NOS inhibitor, L-NAME (30 mg/kg), were used to differentiate between endothelial nitric oxide synthase and nNOS activity [38]. The activity was expressed as pmol/mg protein/min.

### NO assay

The levels of NO (as nitrite) in the penumbral brain area were determined using a Roche’s test “Nitric oxide colorimetric assay” kit. The estimation was based on the following reaction: nitrites + sulfanilamide + *N*-(1-naphthyl)-ethylene-diamine dihydrochloride and yields a reddish-violet diazo dye whose absorbance was measured in the visible range at 540 nm. The assay was performed as described by the manufacturer.

### Statistical evaluation

Statistical analysis was performed using Graph pad prism 5.01 software [39]. Statistical significance was determined using paired or unpaired students’ *t* test as appropriate or one-way ANOVA with Tukey post hoc for three or more groups. Values were expressed as mean ± standard deviation (SD). A *p* value less than 0.05 was considered statistically significant.

## Results

### GSNO inhibits nNOS activity and decreases the levels of neuronal peroxynitrite (3-nitrotyrosine; 3-NT) following IR

**Figure 1:**
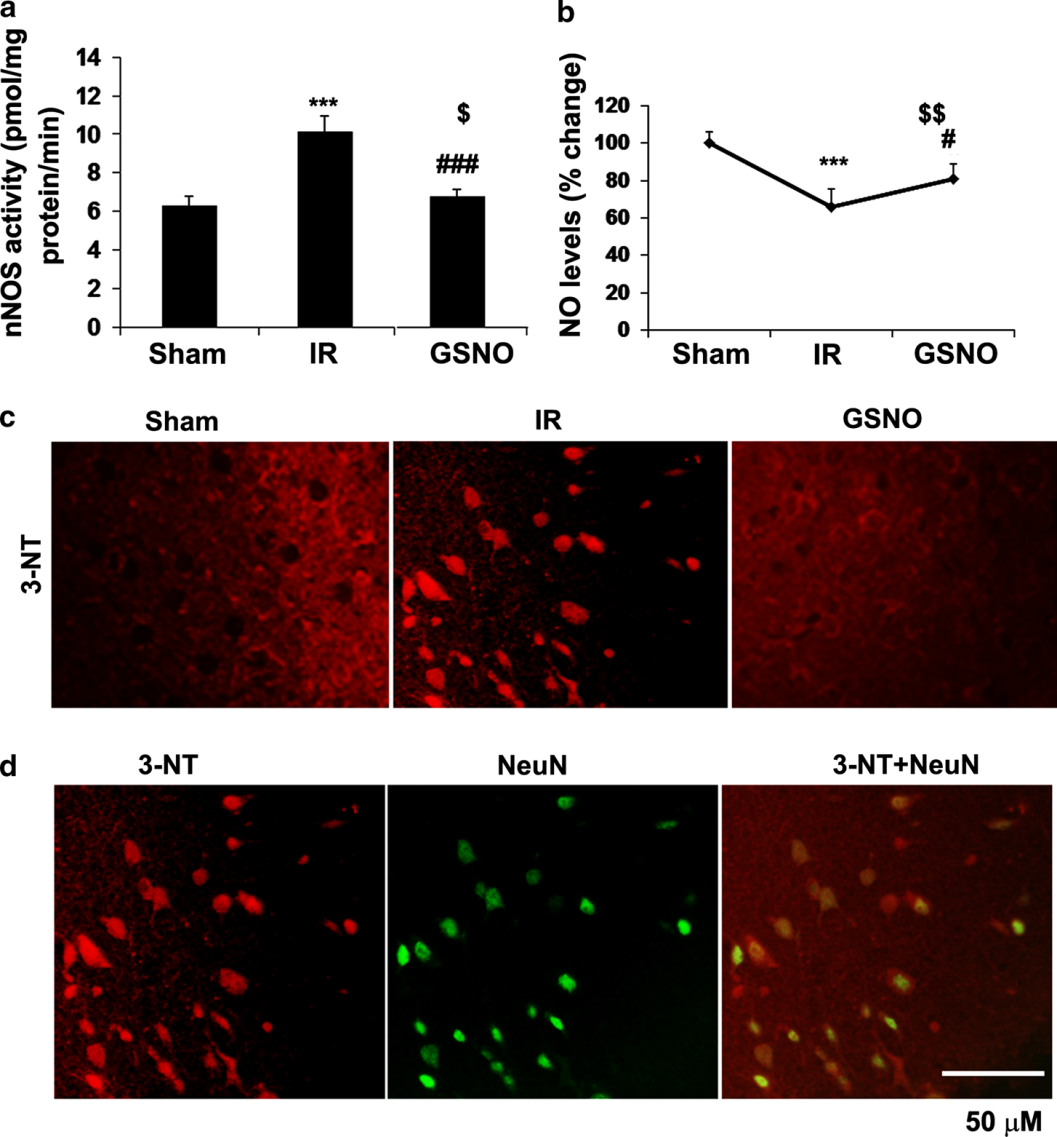
Effect of GSNO on the activity of nNOS and the levels of NO and peroxynitrite (3-NT) in ipsilateral (penumbra) area of the IR brain. nNOS activity at 1 h (**a**), NO levels at 1 h (**b**) and peroxynitrite levels (3-NT, IHC [**c**] and IHC colocalization of 3-NT and neuronal marker NeuN [**d**]) at 1 h after reperfusion were determined. GSNO were administered 0 h after reperfusion began. NO levels in Sham animals were 39.8 ± 4.2 nmol/mg protein. Data are presented as mean ± SD (n = 5). ***p < 0.001 vs. Sham, ^###^p < 0.001, ^#^p < 0.05 vs. IR, ^$$^p < 0.01, ^$^p < 0.05 vs. Sham.

Increased nNOS activity and the participation of nNOS-derived peroxynitrite in stroke injury have been reported [7]. However, the timing and the relationship of peroxynitrite formation with neuronal degeneration are less understood. Furthermore, the mechanisms of nNOS regulation by products of the NO metabolome, including GSNO, are not clear. We observed an increased degree of nNOS activity (p < 0.001) as early as 1 h after reperfusion, and the treatment with GSNO at 0 h after reperfusion significantly decreased (p < 0.001) the nNOS activity (Figure 1a). The increased nNOS activity correlated with the decreased levels (p < 0.001) of NO in the IR brain (Figure 1b), indicating that NO is being consumed by superoxide to make peroxynitrite. Accordingly, enhanced expression of 3-NT (Figure 1c, as a marker of peroxynitrite) was observed in neurons from IR brain (Figure 1d, colocalization of 3-NT with neuronal marker NeuN). GSNO treatment of IR not only increased NO bioavailability (p < 0.05 vs. IR, Figure 1b) but also reduced the expression of 3-NT, thus suggesting that GSNO inhibited nNOS activity. Previously, we have also reported increased levels of 3-NT at 4 h [11] as well as 24 h [26] following IR. These results indicate that IR brain accumulates and maintains sustained levels of peroxynitrite in the acute phase of IR.

### GSNO reduces neuronal degeneration and protects axon/tissue integrity following IR

**Figure 2:**
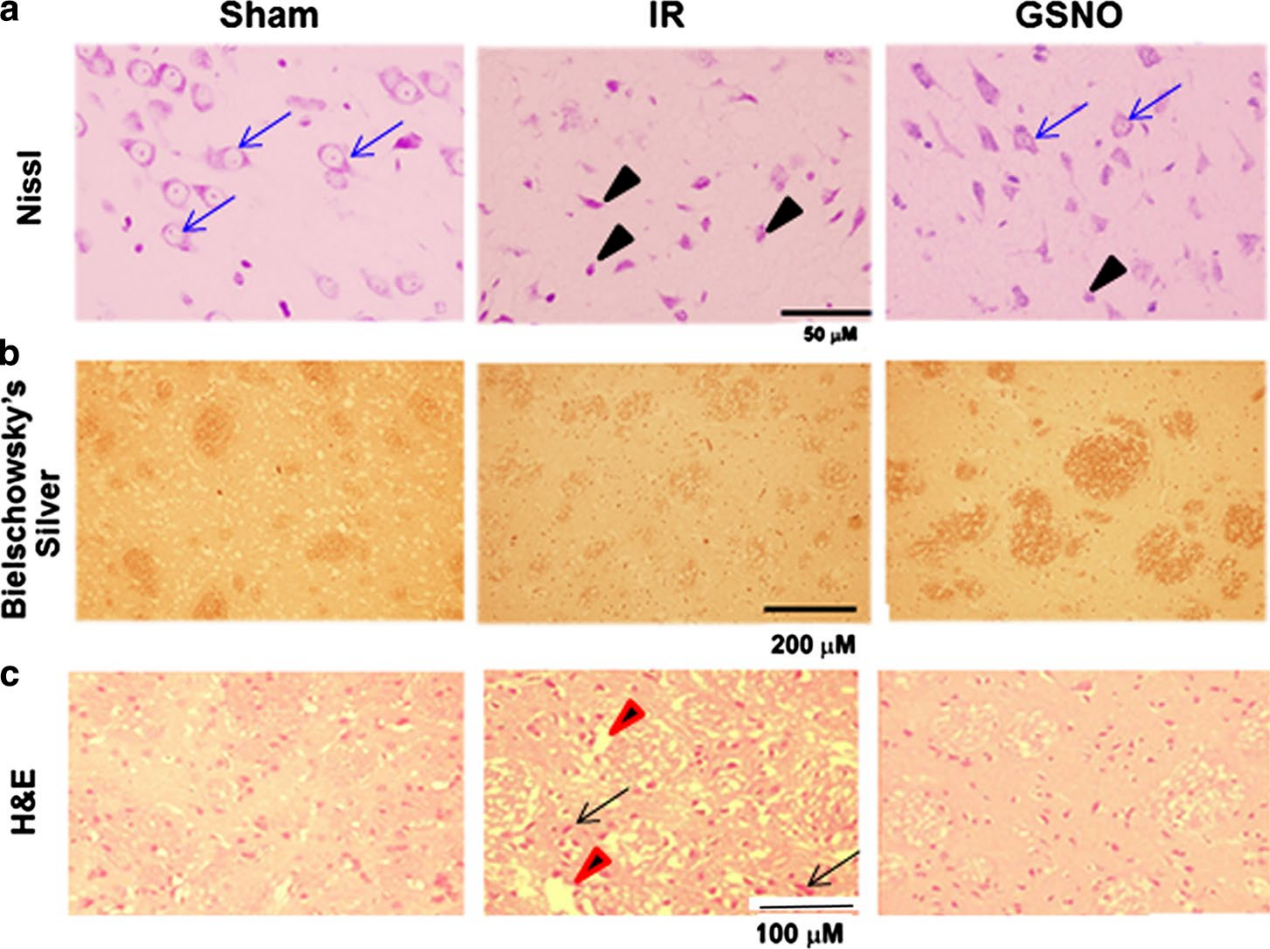
Effect of GSNO on neuronal degeneration and axonal loss in ipsilateral (penumbra) area of IR brain at 4 h after reperfusion. Nissl staining **a** shows significant number of degenerating neurons (*black arrow*) in IR compared with GSNO and sham groups (intact neurons are shown by *blue arrows*). Bielshowsky silver and **b** H&E stainings **c** show loss of axonal and tissue integrity (*red arrow*), respectively, in IR compared with GSNO and sham. IR also shows pyknosis of neurons (*black arrow*). Photomicrographs are representative of n = 3 in each group.

To examine whether GSNO-mediated decreased levels of peroxynitrite in IR brain correlate with neuroprotection, we evaluated neuronal degeneration using Nissl (Figure 2a), axonal integrity using Bielschowsky silver (Figure 2b), and tissue structure using H&E (Figure 2c) in the fixed brain sections at 4 h after IR. Nissl staining showed a remarkable number of degenerating neurons in IR (indicated by black arrow); these levels were reduced in the GSNO group (blue arrow showing intact neurons). Bielschowsky silver staining is a marker of axonal integrity. It was also reduced in IR (Figure 2b). The treatment with GSNO protected axonal integrity, as indicated by the enhanced staining of Bielschowsky silver in the GSNO brain. H&E staining showed that IR-mediated tissue deformation (red arrow) and pyknosis of neurons (black arrow) were blocked by GSNO treatment. These data indicate that GSNO provides neuroprotection.

### GSNO inhibits nNOS activity by decreasing phosphorylation at Ser^1412^ of nNOS following IR

**Figure 3:**
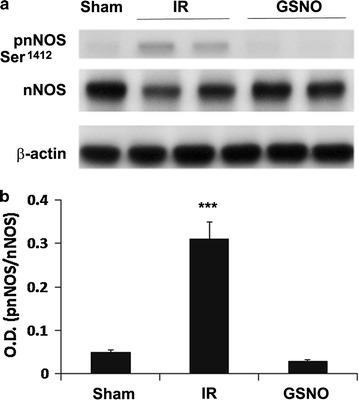
Effect of GSNO on nNOS phosphorylation status in ipsilateral (penumbra) area of IR brain. Phosphorylation at Ser^1417 [equivalent to1412 in human]^ of nNOS and its expression (**a**) and their densitometry (**b**) were evaluated at 1 h of reperfusion. Data are presented as mean ± SD (n = 5). ***p < 0.001 vs. Sham and GSNO.

Immediately after stroke injury, nNOS is activated by NMDA receptor-mediated excitotoxicity and calcium influx through site specific phosphorylation (Ser^1412^) [18]. In such an environment, nNOS-derived NO is converted to peroxynitrite by an instantaneous diffusion limited reaction with superoxide [19]. Peroxynitrite, in turn, maintains aberrant activity of nNOS via oxidative mechanisms. We investigated whether GSNO inhibits nNOS activity as shown in Figure 1a via the reversal of phosphorylation (Ser^1412^). We observed IR-induced increased levels (p < 0.001) of phosphorylated Ser^1412^ at 1 h of reperfusion (Figure 3a, b), resulting in increased nNOS activity as shown in Figure 1a, thus rendering nNOS “aberrant” and causing sustained accumulation of peroxynitrite (Figure 1c). GSNO treatment of IR decreased the levels the IR-induced phosphorylation of Ser^1412^ (p < 0.001, Figure 3a, b), resulting in reduced nNOS activity (Figure 1a) as well as decreased levels of neuronal peroxynitrite (Figure 1c, d).

### nNOS inhibitor 7-NI treatment of IR provides limited protection compared with GSNO following IR

**Figure 4:**
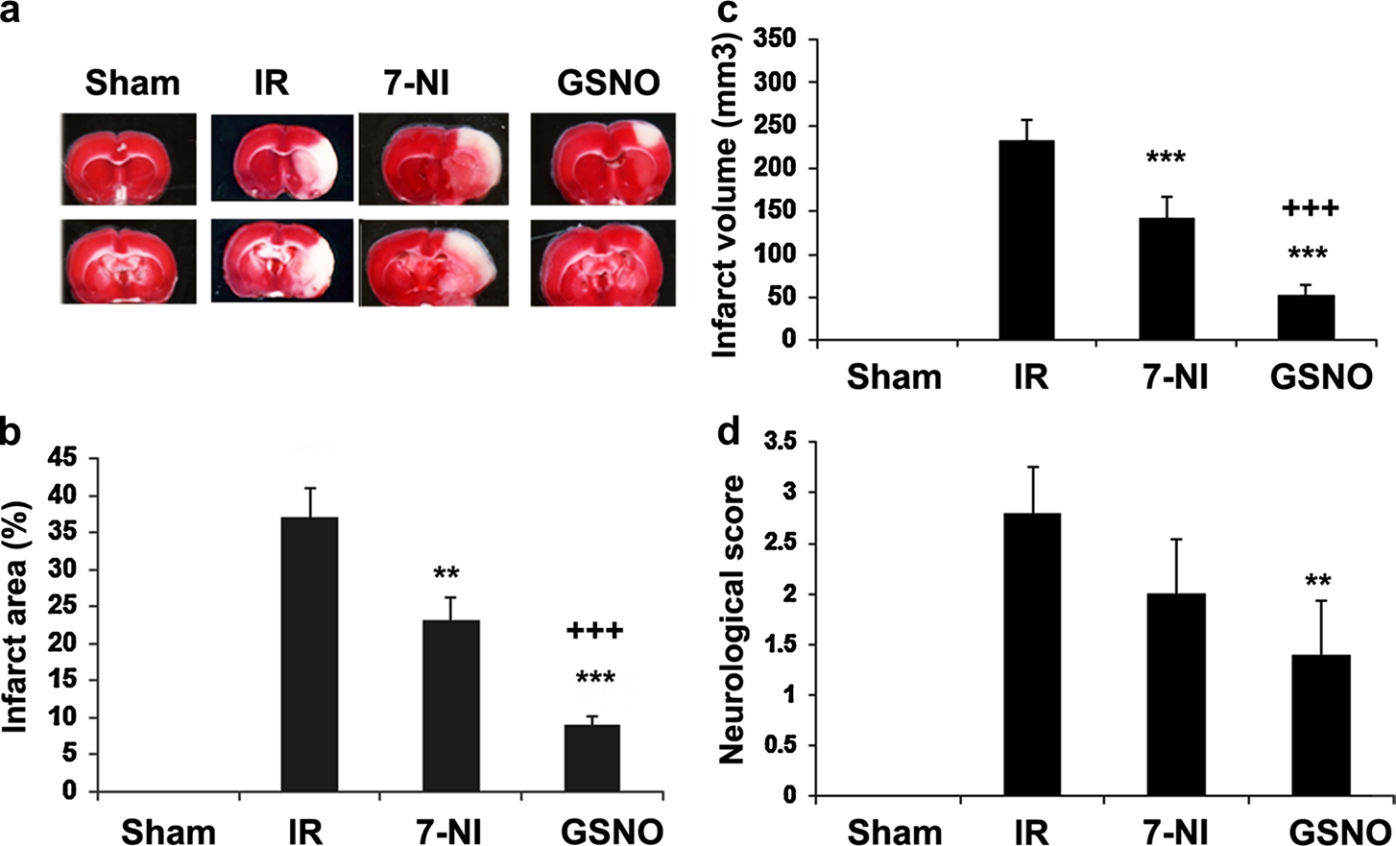
Effect of nNOS inhibitor 7-NI and GSNO on brain infarctions and neurological score. Representative TTC stained sections (#3 and 4 out of six consecutive sections from cranial to caudate region) (**a**), infarct area (**b**), infarct volume (**c**) and neurological score (**d**) at 24 h of the reperfusion after 60 min middle cerebral artery occlusion. Both 7-NI and GSNO were administered 1 h after reperfusion began. Data are presented as mean ± SD (n = 5). ***p < 0.001, **p < 0.01 vs. IR, ^+++^p < 0.001 vs. 7-NI.

7-NI, a selective nNOS inhibitor, is reported to reduce infarct volume in IR models [6, 40]. However, its efficacy is limited due to irreversible nNOS inhibition and a narrow therapeutic window of treatment [40]. We compared the efficacy of 7-NI with GSNO after administering both drugs at 1 h of reperfusion. Treatment with both drugs significantly reduced brain infarctions (p < 0.001, Figure 4a–c) compared with IR, determined at 24 h after IR. However, the efficacy of GSNO was greater than 7-NI in terms of reduction of infarct volume (p < 0.001, Figure 4c). Furthermore, GSNO and not 7-NI significantly improved neurological score (p < 0.01, Figure 4d).

### IR-induced AMPK activation is reversed by GSNO treatment following IR

**Figure 5:**
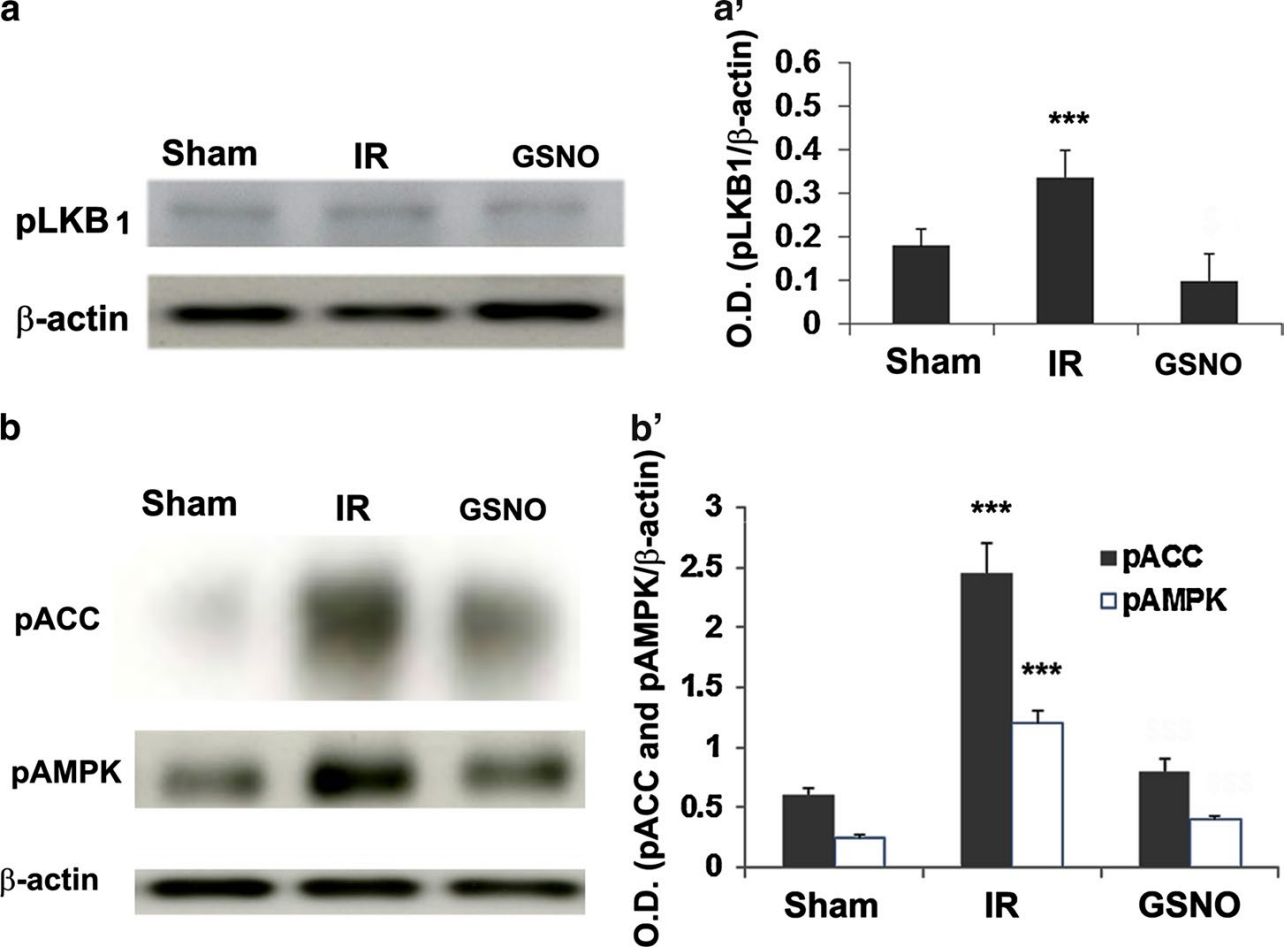
Effect of GSNO on LKB1, ACC and AMPK phosphorylation status in ipsilateral (penumbra) area of IR brain. Western blot at 1 h of reperfusion showing phosphorylation of pLKB1 (**a**, **a**′), pACC and pAMPK/pACC (**b**, **b**′). GSNO was administered at 0 h after reperfusion began. Data are presented as mean ± SD (n = 5). ***p < 0.001, **p < 0.01 vs. Sham and GSNO.

nNOS activation occurs via phosphorylation of Ser^1412^ and thus its increased activity is regulated by several kinases, including Akt and AMPK. While Akt is inhibited [20], AMPK is activated following IR [23]. Peroxynitrite is known to activate AMPK via its upstream kinase LKB1, thus increasing nNOS activity. Consistent with previous reports, we observed IR-induced activation of AMPK (pAMPK), which was blunted by GSNO treatment of IR as shown by western analysis (p < 0.001, Figure 5b, b′) at 1 h after reperfusion. GSNO also inhibited (p < 0.001) IR-induced activation of LKB1, (Figure 5a, a′) and ACC, a substrate of AMPK (Figure 5b, b′).

### AMPK inhibitor Comp c and peroxynitrite scavenger FeTPPS decrease whereas AMPK activator AICAR increases the injury following IR

**Figure 6:**
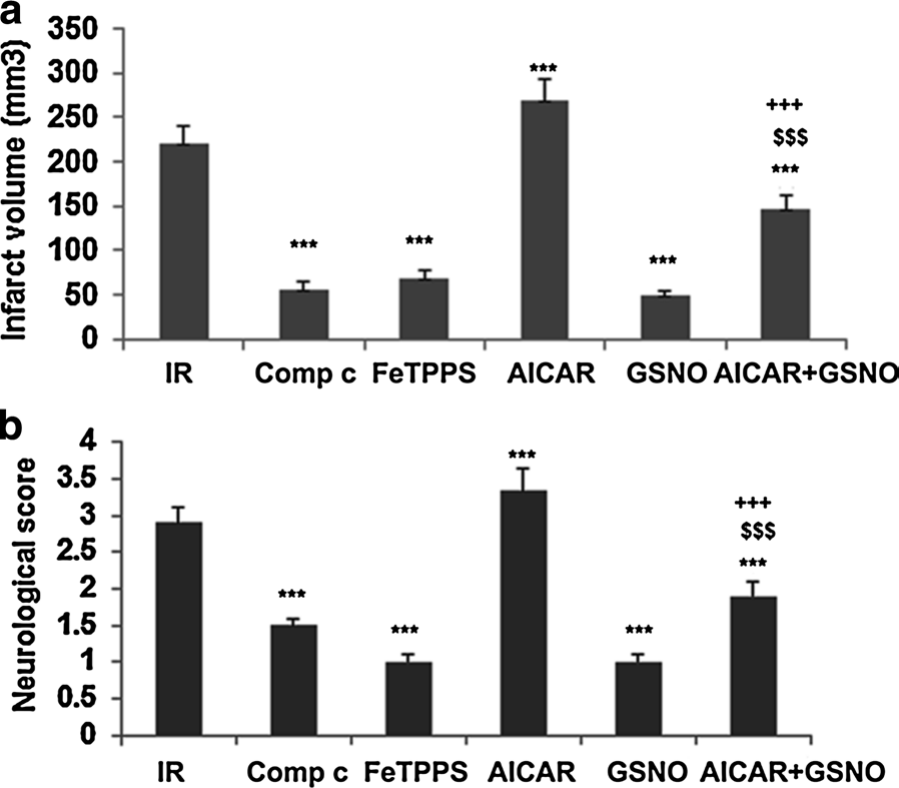
Effect of AMPK inhibitor Comp c, peroxynitrite scavenger FeTPPS and AMPK activator AICAR on infarct volume and neurological score. Infarct volume (**a** calculated from TTC stained sections) and neurological score (**b**) at 24 h of IR. While Comp c, FeTPPS and AICAR were administered 1 h, GSNO was administered 0 h after reperfusion began. Data are presented as mean ± SD (n = 5). ***p < 0.001 vs. IR, ^$$$^p < 0.001 vs. AICAR, ^+++^p < 0.001 vs. GSNO.

AMPK activation in the acute phase of IR is deleterious, and these injurious effects are linked with increased peroxynitrite levels [23, 41]. Peroxynitrite activates AMPK through LKB1activity which, in turn, may phosphorylate nNOS(Ser^1412^)^,^ leading to peroxynitrite formation. We observed that the AMPK inhibitor Comp c as well as the peroxynitrite scavenger FeTPPS-treated animals (at 1 h after IR) had significantly reduced (p < 0.001) infarct volume (Figure 6a) and improved (p < 0.001) neurological score (Figure 6b). In contrast, the AMPK activator AICAR treated animals at 1 h after reperfusion had increased (p < 0.001) levels of infarct volume and a higher (p < 0.001) degree of neurological deficits (Figure 6a, b). However, AICAR animals prior-treated with GSNO (at 0 h of reperfusion) had less (p < 0.001) injury and reduced (p < 0.001) neurological deficits compared with AICAR-alone animals. However, AICAR + GSNO group had significantly more (p < 0.001) infarct volume and neurological deficits. As previously reported [26], GSNO treatment of IR showed remarkable (p < 0.001 vs. IR) neuroprotection and functional recovery (p < 0.001 vs. IR) when administered at reperfusion (Figure 6a, b).

## Discussion

Stroke is one of the most disabling conditions in the United States. The most severe injury after ischemia or reperfusion happens within a couple of hours of initial stroke, leading to the wisdom in stroke care that “time is brain” [42]. This concept demands focus on the initial critical events and their injury mechanisms for developing an effective therapy using clinically relevant animal models.

**Figure 7:**
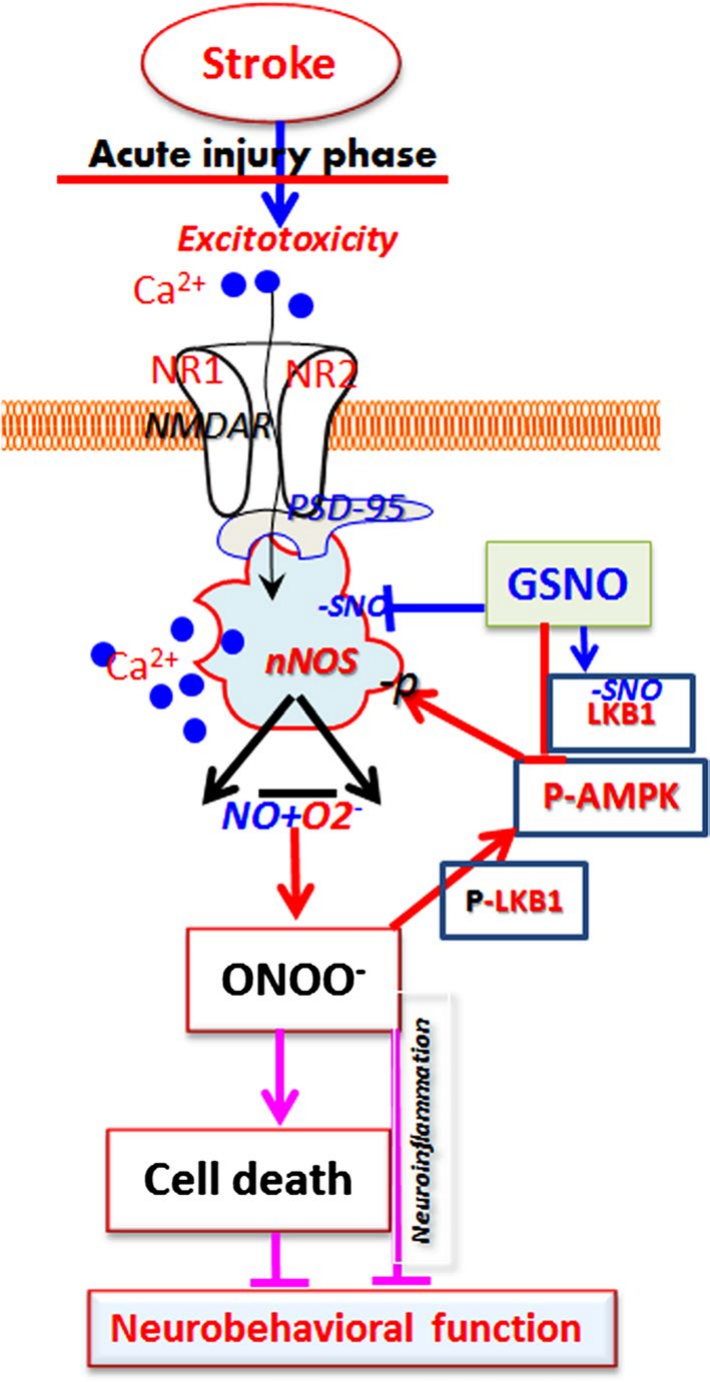
Schematic showing hypothesized nNOS/peroxynitrite-mediated deleterious events and GSNO-mediated targets. IR-induced excitotoxicity and calcium dysregulation cause aberrant nNOS activation, peroxynitrite formation and LKB1/AMPK activation. GSNO treatment of IR blocks the vicious cycle by inhibiting the aberrant activity of nNOS and LKB1/AMPK, leading to neuroprotection and improved neurobehavioral function.

Neuroimaging data in patients who have stroke or transient ischemic attack show that the territory of the middle cerebral artery branches suffers from multiple infarcts more frequently than other areas [43]. Inflammation and secondary brain damage also occur in similar areas after inappropriate thrombolytic therapy [44]. Therefore, we used a rat model of middle cerebral artery occlusion followed by reperfusion. Animal models of focal cerebral ischemia using the middle cerebral artery occlusion technique are recognized as physiologically relevant to stroke in humans [45, 46]. After stroke, the core injury site dies; however, the area surrounding the core (penumbra) survives if treated and perfused properly. Therefore, we selected salvageable penumbra tissue for this study. Our previous studies have shown that the GSNO-mediated mechanisms reduced peroxynitrite levels and inflammation, protected against blood–brain barrier leakage, and improved neurobehavioral function; however, the mechanisms of GSNO-mediated down regulation of peroxynitrite-induced injury in IR are not well understood. nNOS activation due to calcium dysregulation and excitotoxicity has been documented among the causative factors in stroke [18], and the mechanism of S-nitrosylation has been shown to inhibit nNOS activity [17]. Therefore, we hypothesized that GSNO treatment of IR reduces nNOS activity and peroxynitrite levels, leading to neuroprotection and functional recovery as depicted in Figure 7.

Early neuronal cell death in IR is caused by nNOS activation and peroxynitrite formation [7]. Peroxynitrite (3-NT)-positive neurons are more susceptible to cell death [47], indicating that reducing neuronal peroxynitrite may translate into reduced neuronal cell death, leading to tissue protection and functional improvements. In neurons, peroxynitrite originates from excitotoxicity-induced aberrant nNOS activity [7]. nNOS activity is dynamically regulated by NO metabolome, which includes GSNO. NO from nNOS forms GSNO, if superoxide levels are low; however, peroxynitrite is formed when superoxide levels significantly exceed NO levels [48, 49]. Peroxynitrite can keep nNOS under sustained aberrant activation status via the up regulation of phosphorylation of Ser^1412^, thus maintaining its own formation and causing sustained neuronal cell death. In this study, we tested whether GSNO down regulates aberrant nNOS activity by reducing the levels of peroxynitrite through reversing peroxynitrite-induced phosphorylation/dephosphoryaltion mechanisms as elucidated in Figure 7.

nNOS activation requires NMDA-receptor-mediated Ca^2+^ influx, leading to secondary modification of nNOS, such as denitrosylation of Cys^331^, and phosphorylation of Ser^1412^ [50]. Several kinases, including CaMKIIα, PKA, Akt (PKB), and AMPK, and phosphatases, including PP1, PP2A, and PP2B, have been reported to be involved in phosphorylation and dephosphorylation of Ser^1412^ [24, 47, 50–54], but their regulation by peroxynitrite and GSNO is not understood. We observed that IR induced increased levels of phosphorylated Ser^1412^ as early as 1 h following IR (Figure 3). IR also increased nNOS activity (Figure 1a) and neuronal peroxynitrite levels (Figure 1c, d). These IR-induced effects were blunted by GSNO treatment of IR as shown by reduced levels of peroxynitrite (measured as 3-NT expression) (Figure 1c, d) and reduced levels of phosphorylated Ser^1412^ (Figure 3). GSNO also reduces neuronal loss and maintains axonal integrity (Figure 2). Inhibition of nNOS by 7-NI, a selective nNOS inhibitor, has previously been shown to provide neuroprotection as it reduces infarct volume in IR models [40]. However, the efficacy of 7-NI is limited due to irreversible nNOS inhibition and narrow therapeutic window of treatment [6, 40]. We observed reduced infarct volume in 7-NI animals compared with IR; however, GSNO-treated animals had a significantly greater decrease in the infract volume (Figure 4) compared with the 7-NI. GSNO, but not 7-NI, also improved neurological score (Figure 4). Furthermore, 7-NI treatment of IR has been shown to significantly reduce cerebral blood flow [55], indicating that sustained and suicidal inhibition of nNOS may have drastically reduced the NO levels required for blood flow as well as for other NO-dependent physiological function. Contrary to 7-NI, GSNO increases NO bioavailability (Figure 1b), enhances the blood flow and mimics several beneficial functions of NOS-derived NO [26]. Moreover, GSNO maintains the homeostasis of the NO metabolome by regulating nNOS activity in a reversible (feedback) manner via S-nitrosylation and signaling mechanisms through the regulation of its kinases.

Decreased activation of AMPK and reduced IR injury in nNOS KO mice indicate that AMPK activation is dependent on nNOS activity [23]. AMPK activity has been reported to phosphorylate Ser^1412^ of nNOS, thus increasing nNOS activity. Unlike AMPK, Akt is inhibited following IR in a time dependent manner, likely by peroxynitrite [20]. Our data showing decreased activity of LKB1, AMPK and ACC by GSNO indicate that GSNO can reduce nNOS activity through the reversal of AMPK activation (Figure 5). These observations indicate that peroxynitrite, nNOS and AMPK activities participate in a vicious cycle leading to neuronal loss and functional deficits. Multiple lines of evidence indicate that activation of AMPK could result in detrimental outcomes and that restraining AMPK activity confers neuroprotective effects in certain types of brain injury, such as stroke and Alzheimer’s disease [41, 56]. On the one hand, activation of AMPK in response to cellular stress is considered protective and has been established as important in enhancing the lifespan based on studies primarily from non-mammalian systems [57, 58]; additionally, the anti-aging effects of AMPK activation have been proposed to protect against neurodegenerative diseases [59]. It is hypothesized that the metabolic consequences of sustained and aberrant activation of AMPK in the absence of energy deficiency is distinct from those during stress (ATP depletion) conditions. Moreover, the outcome of AMPK activation is dependent on its substrate and the micro environment. Hypoxia/ischemia increases AMPK phosphorylation and the activity which is likely independent of cytosolic AMP levels [23, 41]. IR-mediated deleterious activity of AMPK may result from direct phosphorylation of Thr^172^ by its upstream kinase LKB1. Peroxynitrite has been reported to be potent activator of LKB1/AMPK [60]. In such a case, the ability of AMPK to maintain energy homeostasis would be overwhelmed, and its detrimental effects would in turn be amplified. On the other hand, activation of AMPK in response to therapeutic activation or modest and transient stress, including therapeutic exercises, would likely be protective in restoring the homeostasis of energy metabolism.

Our data on nNOS and AMPK are consistent with the notion that sustained activation of AMPK results in detrimental effects in stroke [23, 41]. AMPK activation leads to the aberrant activation of nNOS, which in turn produces peroxynitrite, the strongest oxidizing agent, causing neuronal cell death. AMPK inhibition, resulting in neuroprotection and reduced levels of peroxynitrite, supports the hypothesis that AMPK activation in the acute phase of stroke is associated with injury. The injurious role of AMPK in the acute phase of IR was supported by our data showing severe injury in AICAR, an AMPK activator, treated animals and reduced injury than IR in compound c, an AMPK inhibitor, treated animals (Figure 6). Significantly reduced infarct volume and decreased functional deficits in peroxynitrite scavenger FeTPPS-treated animals provide further evidence that the deleterious effect of nNOS/AMPK activities was caused by peroxynitrite (Figure 6). Because peroxynitrite activates AMPK via its upstream kinase LKB1 [60] and LKB1 activation can thus be down regulated by GSNO through reducing the levels of peroxynitrite in an nNOS inhibition-dependent pathway, GSNO shows potential to down regulate the nNOS/peroxynitrite/AMPK vicious cycle as depicted in Figure 7.

## Conclusions

In the present study, we have shown that the nNOS/peroxynitrite/AMPK vicious cycle is an attractive therapeutic target for stroke therapy. The study provides evidence that GSNO-mediated mechanisms of phosphorylation and dephosphorylation of nNOS and AMPK, in inhibiting aberrant nNOS and thus reducing peroxynitrite, protected against neuronal loss and aided in functional recovery following IR (Figure 7). GSNO is a natural molecule in the human brain and body, and its exogenous administration has not shown any evident toxicity or side effects in animals and humans [61, 62]. Therefore, GSNO seems to be an ideal interventional drug to provide neuroprotection and stimulate functional recovery in stroke.

## Abbreviations

AICAR: 5-Amino-1-[(2*R*,3*S*,4*R*,5*R*)-tetrahydro-3,4-dihydroxy-5-(hydroxymethyl)furan-2-yl]-1*H*-imidazole-4-carboxamide
AMPK: adenosine monophosphate activated protein kinase
ACC: acetyl-CoA carboxylase
FeTPPS: 5,10,15,20-tetrakis(4-sulfonatophenyl) porphyrinato iron (III)
Compound c: dorsomorphin dihydrochloride
GSNO: S-nitrosoglutathione
H&E: hematoxylin and eosin
IHC: immunohistochemistry
IR: ischemia-reperfusion
LKB: liver kinase B
nNOS: neuronal nitric oxide synthase
NO: nitric oxide
7-NI: 7-nitroindazole
3-NT: 3-nitrotyrosine
Sham: sham-operated animals
